# Quality Improvement Project to Improve COVID-19 Vaccination Uptake on an Old Age Psychiatric Ward (Meridian Ward)

**DOI:** 10.1192/bjo.2022.312

**Published:** 2022-06-20

**Authors:** Fariah Khan, Emma Etherington, Potan Kheder

**Affiliations:** 1 Hammersmith and Fulham Mental Health Unit, London, United Kingdom; 2 Imperial Health Trust, London, United Kingdom

## Abstract

**Aims:**

People with mental health issues have challenges in accessing medical care and it is hypothesised that this may result in a significant number failing to receive protection from COVID -19 by means of vaccination. This study aimed to identify the number of inpatients on a psychiatric general ward (Meridian ward) between May 2021 and June 2021 who were fully vaccinated against COVID-19 so that unvaccinated patients could be offered vaccination during their inpatient stay.

**Methods:**

Data were gathered on the COVID-19 vaccination status of all patients on Meridian ward (inpatient mental health ward) admitted between May and June 2021. This was a total of 10 patients. This information was gathered directly from the patients, their summary care records and GP records. We also audited whether the patient's vaccination status was recorded in the notes.

**Results:**

50% of the patients were fully vaccinated. 20% had received one vaccine and 30% of patients were unvaccinated. 20% of the unvaccinated patients received their first dose during admission.

**Conclusion:**

Patients with mental health issues sufficient to result in psychiatric admission face additional challenges when it comes to receiving a COVID-19 vaccine. It is important that healthcare workers are aware of this and facilitate interventions that maximise vaccination in this at-risk group. To improve uptake among this group it is recommended:

- Patients’ vaccination status is assessed and recorded during inpatient admissions.
- GP surgeries should identify patients on their database with mental health issues who are unvaccinated and providing these individuals with support around accessing information, transport or providing vaccinations at patient's homes.
- There should be clear documentation of vaccination status on summary care record and ward notes.

